# Atrial Remodeling Is Directly Related to End-Diastolic Left Ventricular Pressure in a Mouse Model of Ventricular Pressure Overload

**DOI:** 10.1371/journal.pone.0072651

**Published:** 2013-09-06

**Authors:** Anne Margreet De Jong, Isabelle C. Van Gelder, Inge Vreeswijk-Baudoin, Megan V. Cannon, Wiek H. Van Gilst, Alexander H. Maass

**Affiliations:** 1 Department of Cardiology, University of Groningen, University Medical Center Groningen, The Netherlands; 2 The Interuniversity Cardiology Institute Netherlands, Utrecht, The Netherlands; Scuola Superiore Sant'Anna, Italy

## Abstract

**Background:**

Atrial fibrillation (AF) is often preceded by underlying cardiac diseases causing ventricular pressure overload.

**Objective:**

It was our aim to investigate the progression of atrial remodeling in a small animal model of ventricular pressure overload and its association with induction of AF.

**Methods:**

Male mice were subjected to transverse aortic constriction (TAC) or sham operation. After four or eight weeks, echocardiographic measurements and hemodynamic measurements were made and AF induction was tested. The hearts were either fixed in formalin or ventricles and atria were separated, weighed and snap-frozen for RNA analysis.

**Results:**

Four weeks of pressure overload induced ventricular hypertrophy and minor changes in the atria. After eight weeks a significant reduction in left ventricular function occurred, associated with significant atrial remodeling including increased atrial weight, a trend towards an increased left atrial cell diameter, atrial dilatation and increased expression of markers of hypertrophy and inflammation. Histologically, no fibrosis was found in the left atrium. But atrial gene expression related to fibrosis was increased. Minor changes related to electrical remodeling were observed. AF inducibility was not different between the groups. Left ventricular end diastolic pressures were increased and correlated with the severity of atrial remodeling but not with AF induction.

**Conclusion:**

Permanent ventricular pressure overload by TAC induced atrial remodeling, including hypertrophy, dilatation and inflammation. The extent of atrial remodeling was directly related to LVEDP and not duration of TAC per se.

## Introduction

Atrial fibrillation (AF) is the most common cardiac arrhythmia. Risk factors for AF include hypertension, heart failure, valve disease and advancing age [1]. AF is associated with electrical and structural remodeling, including atrial dilatation, cellular hypertrophy, dedifferentiation, fibrosis, cell death and inflammation [2], [3]. AF mainly occurs in the presence of underlying diseases, with hypertension being the most common [4], [5].

Atrial remodeling is caused by both AF as well as underlying diseases, as has been shown in models of heart failure [6], [7], hypertension [8]–[11], and ventricular pressure overload [11], [12], and also in patients with hypertension, heart failure or valve disease [13]–[16]. Although AF is often preceded by conditions associated with ventricular pressure overload, atrial remodeling in this situation has not been investigated extensively, especially not in small animal models [10]–[12]. The transverse aortic constriction model (TAC) is a well established model in rodents, with left ventricular hypertrophy progressing to heart failure [17], [18]. Our hypothesis was that in this model increased atrial pressure would lead to atrial remodeling accompanied by AF inducibility. Small animal models such as rodents are of interest, as they offer the possibility to investigate the role of specific genes and proteins, i.e. in transgenic and knock-out models, on the development of an AF substrate [19]. In addition, it would offer the opportunity to investigate the effects of therapeutic concepts, such as upstream therapies, on the remodeling process and AF inducibility. Therefore, it was our objective to investigate the progression of atrial remodeling in a mouse model of ventricular pressure overload, representing the situation of associated underlying heart diseases such as hypertension, and to investigate AF inducibility in this model.

## Materials and Methods

### Ethics statement

All experiments were approved by the Committee on Animal Experimentation of the University of Groningen (Approval ID: DEC6121) and were conducted under international guidelines on animal experimentation.

### Animals and housing conditions

Male C57Bl6/J mice at the age of 8–10 weeks, weighing 20–25 grams, were obtained from Harlan (The Netherlands). During the entire experiment, animals were kept on a 12 hr light:12 hr dark cycle with *ad libitum* access to food and water.

### Surgical procedures

Mice were anesthetized using isoflurane (2% in O_2_). TAC was performed as previously described [17]. In short, the thoracic cavity was opened between the 2nd and 3rd rib, and a blunted needle (27G) was placed on the aortic arch between both carotid arteries. The aorta was tied onto the needle with a 7–0 silk suture. Immediately thereafter the needle was removed. Sham operations were performed in the same way, but the suture was removed instead of being knotted. Animals were treated with analgesic medication for 48 hr from the start of operation (Carprofen, 5 mg/kg subcutaneously).

### Echocardiographic measurements

Four or eight weeks after TAC or sham operation, *in vivo* cardiac dimensions and functional parameters were assessed with M-mode and 2D transthoracic echocardiography (Vivid 7 equipped with 14-MHz linear array transducer; GE Healthcare, Chalfont St. Giles, UK). Mice were anesthetized (2% isoflurane in O_2_) as described above, and body temperature was maintained by placing the mouse on a heating pad. Parasternal short axis views were used to record M-mode tracings at left ventricular mid-papillary level to measure left ventricular dimensions (interventricular septum (IVS), left ventricular posterior wall (LVPW), left ventricular internal diameter (LVID)) and % ejection fraction (EF). Apical 4-chamber view was used to assess left atrial (LA) size. LA length was measured at end-ventricular systole from the tip of the mitral valve closure to the base of the LA.

### Hemodynamic measurements

Hemodynamic measurements were carried out prior to sacrifice, four or eight weeks after TAC or sham surgery, using a Millar catheter (Mikro-tip 1.4F; SPR-839, Millar Instruments, Houston, TX, USA). Mice were anaesthetized as described above, and a pressure transducer catheter was inserted via the right carotid artery into the left ventricle. Pressures were recorded in the aorta and subsequently in the left ventricle (LV). Furthermore, in the LV determinants of contraction and relaxation of the LV were determined, i.e. the dP/dtmax and dP/dtmin, indices of maximal contraction and relaxation of the LV, and Tau, an isovolumetric relaxation constant. The corrected dP/dt max and min values were calculated by dividing the dP/dt max and min values by the maximum LV pressure.

### Atrial fibrillation measurements

A surface ECG was recorded using subcutaneous needles to record lead I and lead II positions. Inducibility of atrial arrhythmias was tested by applying a burst of electrical stimuli. Briefly, an octopolar EP cathether 1.1F catheter (Scisense, London, Ontario, Canada) was inserted into the right atrium (RA) via the jugular vein. The catheter was connected to an amplifier (AdInstruments GmbH, Spechbach, Germany) and the signal from the electrodes was monitored using a Powerlab 8/30 (ML870) (AdInstruments GmbH, Spechbach, Germany) and the software LabChart 7 (AdInstruments GmbH, Spechbach, Germany). The inducibility of AF was tested by applying bipolar 5-second bursts of electrical stimuli to the electrodes that reside in the atrium (determined via live monitoring of the signals of the electrodes) using MC_Stimulus II Version 3.2.4 and the stimulator STG4002 (Multi Channel Systems MCS GmbH, Reutlingen, Germany). Atrial capture was confirmed with stimulating at a cycle length of 100 ms. The pulses of the first burst had a cycle length of 50 ms, decreasing to 33 ms, 25 ms, 20 ms, 15 ms and 10 ms in successive bursts. Rectangular pulses of 1 V were used. Between these stimulations a period of 30 s was chosen. AF was defined as a period of rapid irregular atrial rhythm lasting >1s. If 1 or more bursts in the series evoked an AF episode, AF was considered to be inducible. When the AF duration approached 30 s duration the stimulus protocol was manually stopped. The AF episode continued up to a maximum of 5 min, after which the mice was sacrificed.

### Sacrifice

After the hemodynamic measurements, mice were sacrificed by excision of the heart. The heart was either fixed in 4% formalin, desiccated and embedded in paraffin or ventricles and atria were separated, weighed and snap-frozen for RNA analysis.

### Real-time quantitative PCR

Total RNA was isolated from the pooled atria and LV tissue using TRIzol reagent (Invitrogen Corporation, Breda, The Netherlands) and cDNA was synthesized by QuantiTect Reverse Transcription kit (Qiagen, Venlo, The Netherlands) according to the protocol. Gene expression was measured with Absolute QPCR SYBR Green ROX Mix (Abgene, Epsom, United Kingdom) in the presence of 7.5 ng cDNA and 200 nM forward and reverse primers. qRT-PCR was conducted on the Biorad CFX384 (Biorad, Veenendaal, The Netherlands). Initial denaturation and activation of the DNA polymerase at 95°C for 3 min was followed by 35 cycles with denaturation for 15 s at 95°C and annealing and elongation for 30 s at 60°C, followed by a melt curve. Gene expression levels were corrected for ribosomal protein, large, P0 (36b4) reference gene expression, and values were expressed relative to the control group. Primers used are depicted in Table 1.

**Table 1:** Primers.

Gene	Forward	Reverse
**36b4**	aagcgcgtcctggcattgtc	gcagccgcaaatgcagatgg
**Skeletal α-actin**	tgccatgtatgtggctatcca	tccccagaatccaacacgat
**ANP**	atgggctccttctccatcac	tctaccggcatcttctcctc
**BNP**	gggctgtaacgcactgaagt	ggaaagagacccaggcaga
**Collagen type I**	cttcacctacagcacccttgtg	cttggtggttttgtattcgatgac
**Collagen type III**	tcggaactgcagagacctaaa	ccccagtttccatgttacaga
**Fibronectin**	agaccatacctgccgaatgtag	gagagcttcctgtcctgtagag
**MMP2**	ccctgatgtccagcaagtag	ggagtctgcgatgagcttag
**TIMP1**	ctgctcagcaaagagctttc	ctccagtttgcaagggatag
**GDF15**	tgacccagctgtccggatac	gtgcacgcggtaggcttc
**IL-6**	ctggtcttctggagtaccatag	tccttagccactccttctgt
**MCP-1**	accagcagcaggtgtccc	gcacagacctctctcttgagctt
**Rcan1**	gcttgactgagagagcgagtc	ccacacaagcaatcagggagc
**LTCC**	agatccagccatctccaaagag	ccttttgtcgctttagacattcc
**Kcnd2**	tagaggcagtgtgcaagaac	attgctgtggtcacgtaagg
**Kcnd3**	acacctgcccaactctaac	cagtccatcgtctgctttc
**Kcnq1**	gcaaagaccgtggcagtaac	atggacagcagctggtggag
**Kcnj2**	cggctcattctctttcac	atggatgcttccgagaac
**Kcnj3**	ctggaaggcattgtggaaac	gcatggaactgggagtaatc

36b4, ribosomal protein, large, P0; ANP, atrial natriuretic peptide; BNP, brain natriuretic peptide; MMP2, matrix metalloproteinase-2; TIMP1, tissue inhibitor of metalloproteinase 1; GDF15, growth differentiation factor 15; IL-6, interleukin-6; MCP-1, monocyte chemotactic protein 1; Rcan1, regulator of calcineurin 1. LTCC, α1c subunit voltage dependent L-type calcium channel; Kcnd2, potassium voltage-gated channel, Shal-related family, member 2; Kcnd3, potassium voltage-gated channel, Shal-related family, member 3; Kcnq1, potassium voltage-gated channel, subfamily Q, member 1; Kcnj2, potassium inwardly-rectifying channel, subfamily J, member 2; Kcnj3, potassium inwardly-rectifying channel, subfamily J, member 3.

### Histology

Tissue was cut in 4 μm sections. To determine the amount of fibrosis, a Masson trichrome staining was performed as described before [20]. Sections were photographed at the x40 scanning mode with a slidescanner (NanoZoomer 2.0-HT, Hamamatsu Photonics Nederland, Almere, the Netherlands) and analyzed with Image Scope 11 (Aperio Technologies, Inc. Vista, CA, USA). Fibrosis was expressed as the percentage of blue pixels compared to the total area. The edge of the tissue was excluded. To measure cardiomyocyte diameter a lectin staining was performed to detect cell membranes, a counterstaining with DAPI (Vector Laboratories, Burlingame, CA, USA) was done to see the nuclei. Sections were photographed and cell diameter was determined using Image J (Image J 1.43u, NIH, USA). Cardiomyocytes that were included were transversally cut and a nucleus could be seen. Per animal 5 different fields were used and on average 60–65 cells were measured. For histology of the LV, short-axis sections were used, for analysis of the atria the hearts were cut in the long-axis orientation.

### Statistics

Data are expressed as mean values ± standard error of the mean (SEM). Comparisons between groups were done using one-way analysis of variance (ANOVA) with post hoc Tukey's test. Correlations were analyzed using Pearson *r* correlation coefficient. All analyses were done using IBM SPSS Statistics (Version 20, SPPS Inc., Chicago, IL, USA). P-values of <0.05 were considered statistically significant.

## Results

### Validation of the model – ventricular remodeling

**Figure 1:**
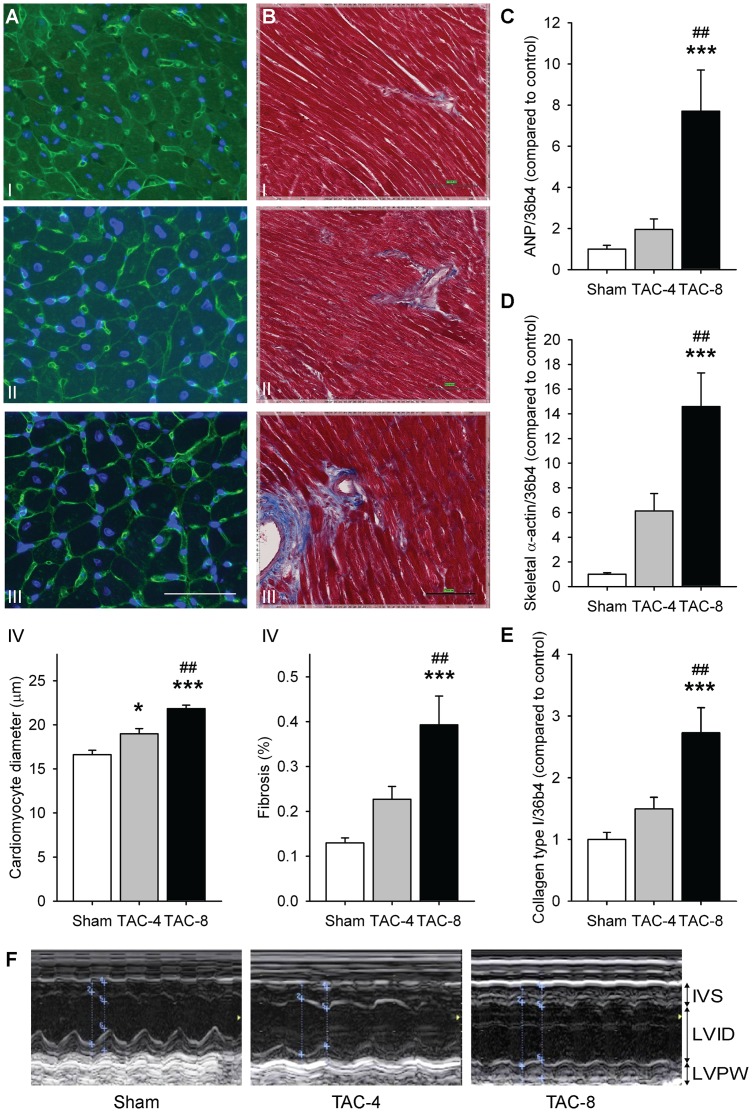
In the left ventricle, ventricular pressure overload induced hypertrophy and fibrosis. A) Cell diameter (n = 7–8 per group) – I sham, II TAC 4 weeks, III TAC 8 weeks, IV quantification -, the ruler is 50 μm, B) Masson's trichrome staining (n = 13–18 per group) – I sham, II TAC 4 weeks, III TAC 8 weeks, IV quantification -, the ruler is 100 μm, and C) mRNA expression of ANP (n = 12–17 per group), D) skeletal α-actin (n = 12–17 per group) and E) collagen type I (n = 12–17 per group). F) representative echocardiographic images. *P<0.05, **P<0.01, ***P<0.001 vs sham. ^#^P<0.05, ^# #^P<0.01, ^# # #^P<0.001 vs TAC 4 weeks. ANP, atrial natriuretic peptide.

Table 2 shows changes in LV hemodynamic parameters, echocardiographic parameters and organ weights after four and eight weeks of ventricular pressure overload. There was a significant increase in aortic and ventricular pressure, ventricular wall thickness and heart weight and a reduction of LV ejection fraction. This was accompanied by an increased left ventricular cell diameter (Figure 1a) and induction of fibrosis (Figure 1b). In addition, left ventricular gene expression associated with hypertrophy was increased: 2.0-fold and 7.1-fold increase in atrial natriuretic peptide (ANP) (Figure 1c), 2.0-fold and 3.5-fold increase in brain natriuretic peptide (BNP) (Table 2), 1.6-fold and 9.4-fold increase in GDF15 (Table 2), 6.1-fold and 14.6-fold increase in skeletal α-actin (Figure 1d) and 1.9-fold and 6.4-fold increase in β-myosin heavy chain (β-MHC) (Table 2), after four and eight weeks of TAC, respectively. Furthermore, expression of genes associated with fibrosis was increased, including expression of collagen I (Figure 1e), collagen III and fibronectin (Table 2). Figure 1f shows representative echocardiographic images.

**Table 2:** Hemodynamic parameters, echocardiographic parameters, organ weights and left ventricular gene expression.

	Sham	TAC – 4 weeks	TAC – 8 weeks
**Hemodynamic parameters**			
Maximum pressure aorta (mmHg)	99.97±2.20	130.85±2.38***	144.46±4.66***^#^
Maximum pressure LV (mmHg)	101.02±2.42	126.63±2.94***	140.00±53***^#^
dP/dtmax (mmHg/s)	8343.2±320.9	7032.7±200.4*	7627.1±339.7
dP/dtmin (mmHg/s)	−7771.8±407.1	−6550.2±327.6	−6860.1±470.7
Corrected dP/dtmax	82.99±3.09	55.71±1.56***	54.44±1.60***
Corrected dP/dtmin	−77.21±3.88	–51.83±2.50***	−48.83±2.78***
Tau (ms)	5.83±0.38	8.60±0.62**	8.66±0.77**
**Echocardiographic parameters**			
IVSd (mm)	0.71±0.02	0.97±0.03***	1.00±0.03***
LVPWd (mm)	0.71±0.01	1.01±0.03***	1.01±0.03***
LVIDd (mm)	3.82±0.05	3.75±0.10	4.28±0.09***^###^
LVIDs (mm)	2.23±0.05	2.55±0.09	3.34±0.16***^###^
EF (%)	78.41±1.02	66.63±1.80**	49.81±4.13***^###^
**Weight**			
Body weight (g)	27.91±0.53	26.55±0.56	27.71±0.36
Heart weight (mg/mm)	8.56±0.09	10.25±0.36*	12.74±0.58***^###^
LV weight (mg/mm)	6.31±0.08	7.61±0.22*	10.68±0.52***^###^
RV weight (mg/mm)	1.65±0.04	1.77±0.11	2.02±0.13*
Lung weight (mg/mm)	10.06±0.30	11.08±0.50	12.39±1.01*
Kidney weight (mg/mm)	23.05±0.35	21.37±0.48	21.07±0.72*
Liver weight (mg/mm)	75.71±2.31	73.17±2.47	73.31±1.48
**LV mRNA expression**			
BNP/36b4	1.00±0.11	2.05±0.19	3.50±0.63***^#^
GDF15/36b4	1.00±0.11	1.62±0.19	9.39±3.74**^#^
β-MHC/36b4	1.00±0.07	1.89±0.25	6.36±1.48***^##^
Collagen type III/36b4	1.00±0.07	1.67±0.17	2.31±0.36***
Fibronectin/36b4	1.00±0.07	1.58±0.18	2.29±0.50**

Echocardiographic measurements (n = 15–25 per group), hemodynamic measurements (n = 15–21 per group), heart, liver, long and kidney weights (15–25 per group), LV and RV weight (n = 11–16 per group) and left ventricular mRNA expression of genes associated with hypertrophy and fibrosis (n = 12–17 per group). Organ weights are corrected by tibia length. Results are expressed as mean ± SEM. Gene expression data is shown compared to control. *P<0.05, **P<0.01, ***P<0.001 vs sham. ^#^ P<0.05, ^# #^P<0.01, ^# # #^P<0.001 vs TAC 4 weeks. TAC, transverse aortic constriction; IVSd, diastolic interventricular septum; LVPWd, diastolic left ventricular posterior wall; LVIDd, diastolic left ventricular internal diameter; LVIDs, systolic left ventricular internal diameter; EF, ejection fraction; LV, left ventricle; RV, right ventricle; BNP, brain natriuretic peptide; GDF15, growth differentiation factor 15; β-MHC, β-myosin heavy chain.

### Atrial remodeling

**Figure 2:**
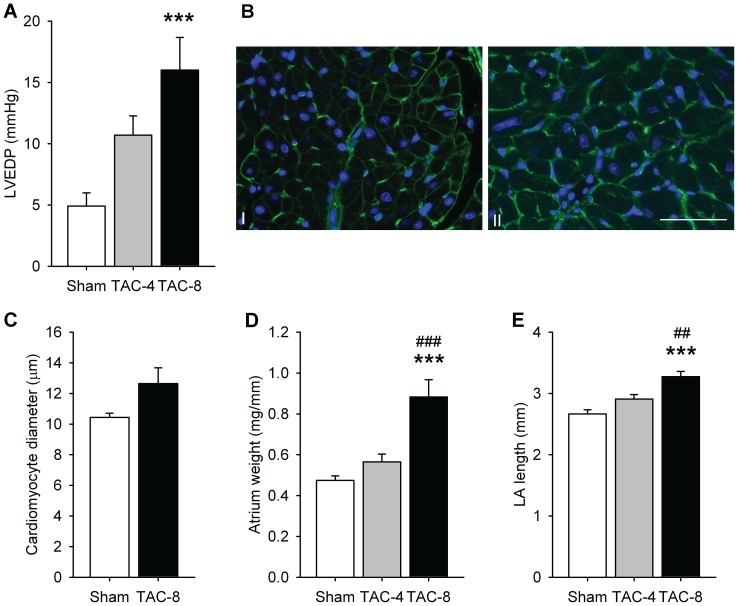
Ventricular pressure overload increased LVEDP and induced atrial hypertrophy and atrial dilatation. A) LVEDP (n = 15–21 per group), B) lectin staining – I sham, II TAC 8 weeks -, the ruler is 50 μm, C) quantification of the lectin staining (n = 6–7 per group), D) increased atrial weight, corrected by tibia length (n = 11–16 per group) and E) increased LA length (n = 14–23 per group). *P<0.05, **P<0.01, ***P<0.001 vs sham. ^#^P<0.05, ^# #^P<0.01, ^# # #^P<0.001 vs TAC 4 weeks. LVEDP, left ventricle end diastolic pressure.

TAC caused an increased LV end diastolic pressure (LVEDP) (Figure 2a). In the atria, we observed a trend towards an increase in cell diameter in the LA after eight weeks (p = 0.08, Figure 2b–c). This was accompanied by an increased weight of the combined atria (Figure 2d) and LA length as measured by echocardiography (Figure 2e).

**Figure 3:**
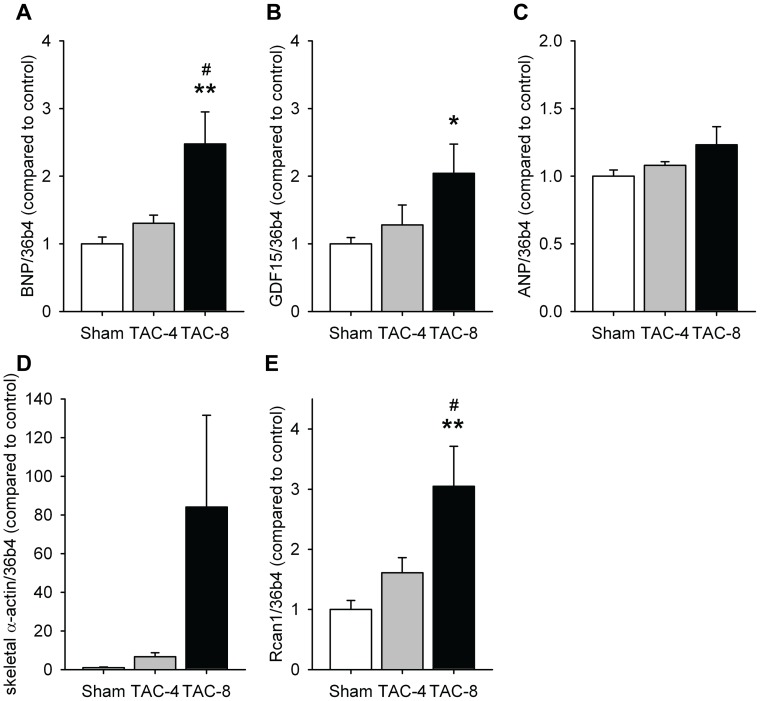
Atrial mRNA expression levels related to hypertrophy were increased. A) ANP (n = 12–16 per group), B) BNP (n = 12–16 per group), C) GDF15 (n = 12–16 per group), D) skeletal α-actin (n = 12–13), E) Rcan1 (n = 12–16 per group). *P<0.05, **P<0.01, ***P<0.001 vs sham. ^#^P<0.05, ^# #^P<0.01, ^# # #^P<0.001 vs TAC 4 weeks. ANP, atrial natriuretic peptide; BNP, brain natriuretic peptide; GDF15, growth differentiation factor 15; Rcan1, Regulator of calcineurin 1.

Gene expression markers, as determined in pooled atria, of atrial hypertrophy were increased, including BNP (Figure 3a) and growth differentiation factor 15 (GDF15) (Figure 3b). ANP expression was not significantly increased. Skeletal α-actin (Figure 3d), a marker of hypertrophy and dedifferentiation was increased, although not significantly. Expression of regulator of calcineurin (Rcan1), an indication of calcineurin activation, was increased (Figure 3e).

**Figure 4:**
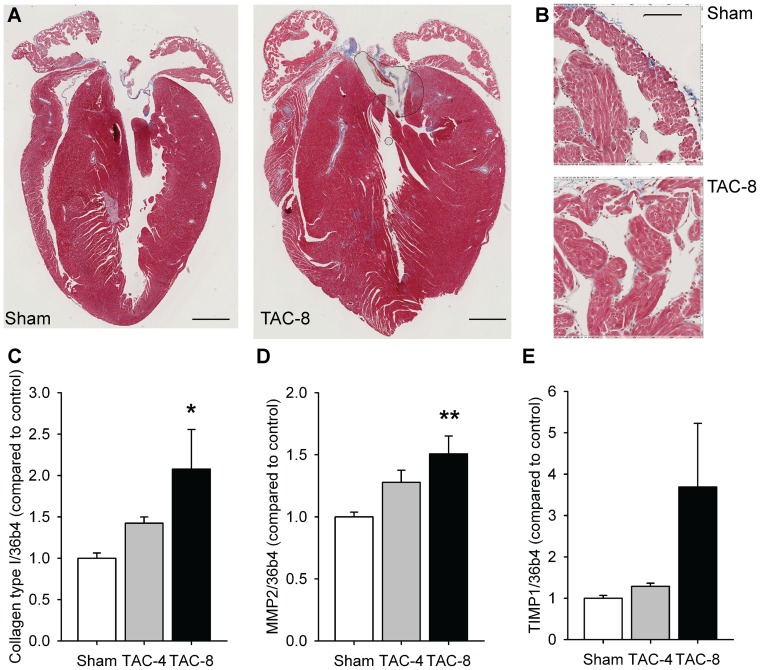
In the atria ventricular pressure overload was not clearly associated with fibrosis. A) Masson's trichrome staining, the ruler is 1 mm, B) LA close-up of Masson's trichrome staining, the ruler is 100 μm, C) mRNA expression of collagen type I (n = 12–16 per group), D) MMP2 (n = 12–17 per group) and E) TIMP1 (n = 12–17 per group. *P<0.05, **P<0.01, ***P<0.001 vs sham. ^#^P<0.05, ^# #^P<0.01, ^# # #^P<0.001 vs TAC 4 weeks. MMP2, matrix metalloproteinase-2; TIMP1, tissue inhibitor of metalloproteinase 1.

Increased fibrosis in the LA was not detected in a Masson staining (Figure 4a–b), but atrial expression of collagen type I (Figure 4c), collagen type III (1.6-fold [ns] and 2.1-fold [P<0.01]) and fibronectin (1.6-fold [ns] and 2.7-fold [P<0.05]) were increased. Expression of MMP2 and TIMP1 (trend) (Figure 4d, e) was increased after 8 weeks of TAC, expression of TIMP2, 3 and 4 was unchanged as well as expression of MMP14.

**Figure 5:**
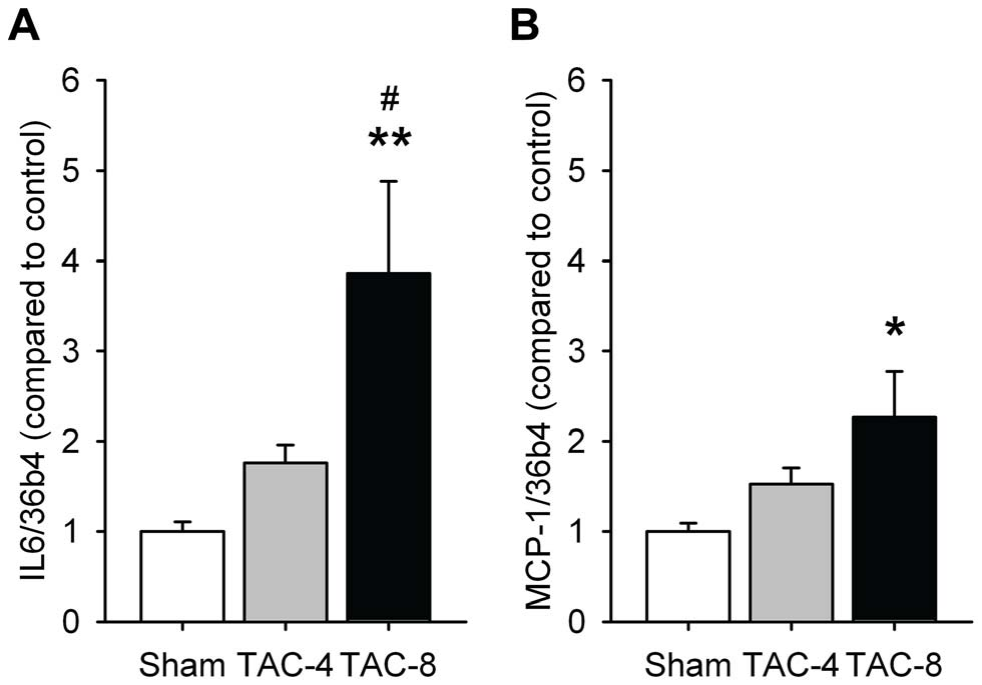
Ventricular pressure overload increased atrial mRNA expression of markers related to inflammation. A) IL-6 (n = 12–16 per group) and B) MCP-1 (n = 12–16 per group). *P<0.05, **P<0.01, ***P<0.001 vs sham. ^#^P<0.05, ^# #^P<0.01, ^# # #^P<0.001 vs TAC 4 weeks.

Inflammation was shown by increased expression of the inflammatory cytokines interleukin-6 (IL-6) and monocyte chemoattractant protein 1 (MCP-1) after eight weeks of pressure overload, by 3.9-fold and 2.3-fold, respectively (Figure 5).

**Figure 6:**
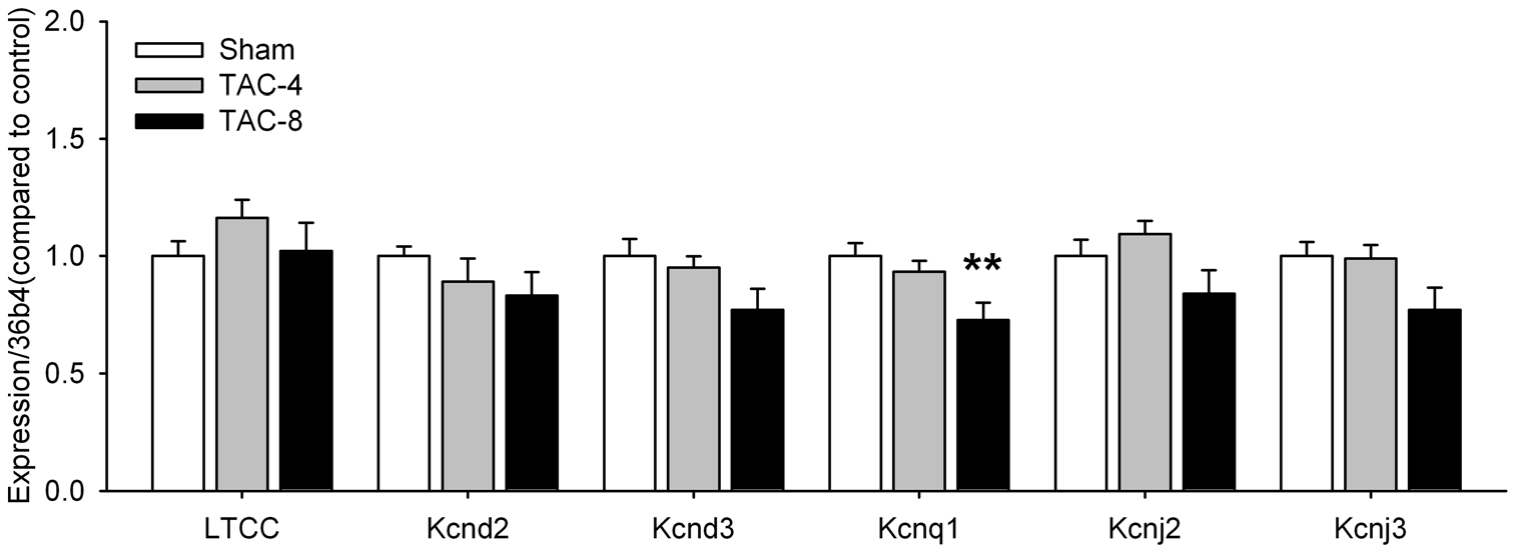
Expression levels of ion channels related to electrical remodeling. (n = 12–16 per group). *P<0.05, **P<0.01, ***P<0.001 vs sham. LTCC, α1c subunit voltage dependent L-type calcium channel; Kcnd2, potassium voltage-gated channel, Shal-related family, member 2; Kcnd3, potassium voltage-gated channel, Shal-related family, member 3; Kcnq1, potassium voltage-gated channel, subfamily Q, member 1; Kcnj2, potassium inwardly-rectifying channel, subfamily J, member 2; Kcnj3, potassium inwardly-rectifying channel, subfamily J, member 3.

No major changes in gene expression related to electrical remodeling were observed. Figure 6 shows no change in gene expression of the L-type calcium channel. Although expression levels of Kcnd2, Kcnd3, Kcnj2 and Kcnj3 were reduced, this was not significant. These genes encode Kv4.2, Kv4.3, Kir2.1 and Kir3.1 and contribute to the cardiac transient outward potassium current (*I*_to_), inward rectifier current (*I*_K1_) and acetylcholine-activated inward rectifier K^+^ current *(I*_KACh_). Expression of Kcnq1, which encodes Kv7.1 and mediates the slow delayed rectifying potassium current (*I*_Ks_), was reduced to 73% of control levels in animals subjected to TAC for 8 weeks.

### Atrial fibrillation

**Figure 7:**
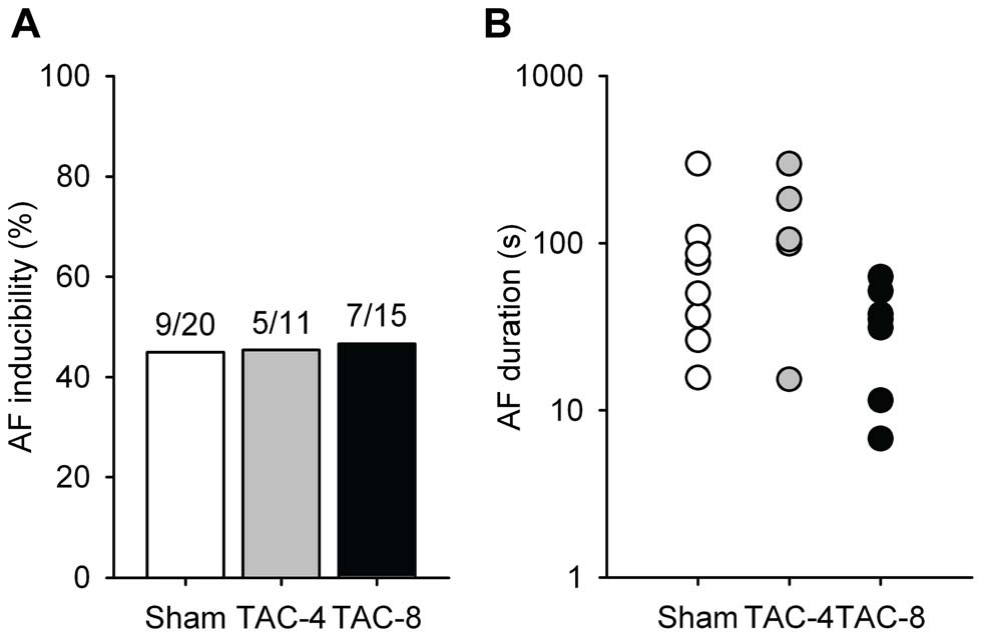
AF inducibility and duration in mice subjected to sham surgery or ventricular pressure overload. A) AF inducibility and B) Duration of the longest AF episode in each mouse.

AF induction was comparable between the groups, both in incidence and duration (Figure 7).

### Correlations between LVEDP and markers of atrial remodeling

**Figure 8:**
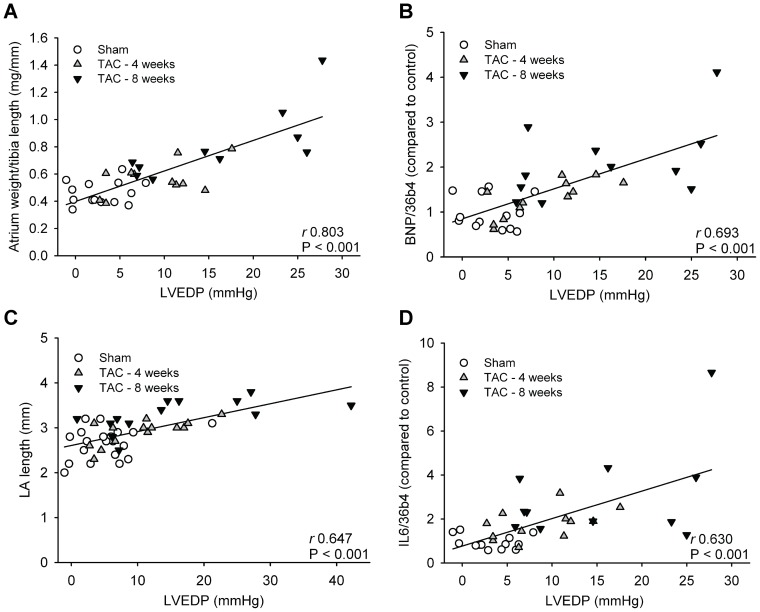
Correlations between LVEDP and parameters of atrial remodeling. A) Atrial weight (n = 35), B) BNP expression (n = 36), C) LA length as determined via echocardiography (n = 47) and D) IL6 expression (n = 36). LVEDP, left ventricular end diastolic pressure; BNP, brain natriuretic peptide; IL6, interleukin 6.

Figure 8 shows that there is a correlation between LVEDP and markers of atrial remodeling, as determined in the combined atria, independent of duration of TAC. LVEDP correlated with markers of atrial hypertrophy (weight of the combined atria, BNP expression), dilatation (LA length) and inflammation (IL-6). Table 3 shows that also other markers of remodeling correlated with LVEDP and with atrial weight. Interestingly, although LA fibrosis was not increased using Masson's staining, expression levels of collagen type I, collagen type III, fibronectin, MMP2 and TIMP1 did correlate with LVEDP and atrial weight. For genes related to electrical remodeling, there was no correlation with LVEDP and a weak correlation with atrial weight.

**Table 3:** Correlations – Pearson *r*.

	LVEDP		Weight	
Genes	*r*	p-waarde	*r*	p-waarde
ANP	0.569	<0.001	0.646	<0.001
BNP	0.693	<0.001	0.695	<0.001
GDF15	0.559	<0.001	0.741	<0.001
Skeletal α-actin	0.357	0.045	0.534	0.001
Rcan1	0.659	<0.001	0.556	<0.001
Collagen type I	0.509	0.002	0.775	<0.001
Collagen type III	0.586	<0.001	0.759	<0.001
Fibronectin	0.533	<0.001	0.807	<0.001
MMP2	0.655	<0.001	0.640	<0.001
TIMP1	0.478	0.003	0.759	<0.001
IL6	0.630	<0.001	0.722	<0.001
MCP-1	0.348	0.037	0.429	0.007
LTCC	0.377	0.023	0.189	0.255
Kcnd2	−0.072	0.678	−0.448	0.005
Kcnd3	−0.198	0.246	−0.382	0.018
Kcnq1	−0.224	0.190	−0.412	0.010
Kcnj2	−0.024	0.890	−0.075	0.653
Kcnj3	−0.109	0.528	−0.455	0.004
LA length	0.647	<0.001	0.387	0.029
Atrial weight	0.803	<0.001		

ANP, atrial natriuretic peptide; BNP, brain natriuretic peptide; GDF15, growth differentiation factor 15; Rcan1, regulator of calcineurin; MMP2, matrix metalloproteinase-2; TIMP1, tissue inhibitor of metalloproteinase 1; IL-6, interleukin-6; MCP-1, monocyte chemoattractant protein 1; LTCC, α1c subunit voltage dependent L-type calcium channel; Kcnd2, potassium voltage-gated channel, Shal-related family, member 2; Kcnd3, potassium voltage-gated channel, Shal-related family, member 3; Kcnq1, potassium voltage-gated channel, subfamily Q, member 1; Kcnj2, potassium inwardly-rectifying channel, subfamily J, member 2; Kcnj3, potassium inwardly-rectifying channel, subfamily J, member 3; LA, left atrium.

## Discussion

The present study showed that four weeks of pressure overload induced ventricular hypertrophy and only minor changes in the atria. After eight weeks of pressure overload, a significant reduction in left ventricular function occurred, associated with significant atrial remodeling including increased atrial weight, a trend towards an increased LA cell diameter, LA dilatation and increased expression of markers of hypertrophy and inflammation. Minor changes related to electrical remodeling were observed. In addition, a significant increase in atrial fibrotic gene expression without histologically detectable fibrosis in the LA was observed. The extent of atrial remodeling was directly related to LVEDP and not to duration of TAC per se.

This study was designed to investigate atrial remodeling occurring in diseases preceding AF. In large animal models of hypertension, atrial remodeling including atrial dilatation, cellular hypertrophy, fibrosis and inflammation and increased AF inducibility have been described [8], [9]. However, small animal models are more useful to investigate the effect of specific genes on the AF substrate [19]. Although a range of mouse models related to AF have been described, the vast majority are transgenic models [21]. Therefore, in this study a model of TAC was used causing a sudden onset of pressure overload of the heart to mimic the situation preceding AF, caused by underlying diseases such as hypertension. This model has not been used extensively to investigate atrial remodeling. Liao *et al.* demonstrated increased atrial weight after 10 days of TAC, which was accompanied by increased fibrosis and inflammation and increased inducibility of AF, albeit short episodes [12]. Other studies of pressure overload in rodents also showed an increase in atrial diameter [22], atrial cellular hypertrophy [23] and an increase in atrial weight, which was, as in our mice related to the severity of left ventricular dysfunction [24]. These studies, however, did not investigate AF induction [22]–[24]. In rats, different models of pressure overload showed increased atrial remodeling which was associated with increased AF inducibility [10], [11], [25], [26].

Compared to these studies, we observed similar adaptation processes occurring in the atria in response to the increased ventricular pressure but without an increased inducibility of AF. We could demonstrate atrial hypertrophy as was shown by an increased atrial weight, increased expression of genes associated with hypertrophy and a trend towards an increase in LA cell diameter. This trend and lack of significance in increase of atrial cell diameter is possibly due to the low number, as atrial cell diameter did correlate with the total heart weight. Interestingly, ANP, a marker often used for ventricular hypertrophy was not significantly increased in the atria, most likely due to the already high baseline expression levels of ANP in the atria (75-fold higher in the atria compared to the ventricles). In addition, we observed an increased expression of markers of inflammation. Only minor changes related to electrical remodeling were observed. Although we observed an increased expression of genes associated with fibrosis, as well as of some MMP and TIMPs, we could not detect increased fibrosis histologically.

In contrast to our expectations AF inducibility was similar in sham operated animals and after pressure overload for four and eight weeks. The similar levels of AF inducibility might be explained by the lack of histologically detectable fibrosis, which is known to promote reentry and thus AF, or the lack of major changes in ion channel expression, which has been related to electrical remodeling. Another explanation might be the relatively high level of AF inducibility in the sham operated animals, possibly related to the thoracotomy. In humans, postoperative AF occurs relatively often after cardiac surgery. For coronary artery bypass surgery the reported incidence varies from 16–50% [27].

Of most interest is the correlation between LVEDP and total atrial weight with markers of atrial remodeling, showing increased atrial remodeling with increased LVEDP and atrial weight. Some animals with four weeks TAC had higher LVEDPs than those with eight weeks TAC (Figure 8) also demonstrating more extensive remodeling. Increases in LA pressure through ventricular pressure overload therefore seems to be more important for remodeling than duration of the hemodynamic alterations.

The use of mice in genetic models offers the advantage that methods are established and could therefore also be used to investigate atrial remodeling [19]. For example, Reil et al. showed pronounced atrial remodeling and AF inducibility in mice with cardiac overexpression of Rac1 [28], other examples include cardiac specific overexpression of ACE and TGFβ1 which also show atrial remodeling and increased AF inducibility [29], [30]. Many more transgenic mouse models have been described related to AF [21]. However, to investigate the effects of proteins with possible protective roles, a model with pronounced atrial remodeling should be used. At this moment, this is, for example, investigated in drosophila subjected to tachypacing [31]. A major drawback of drosophila is that these are invertebrates and their heart consists of a single tube. It would be useful to have a non-genetic model to investigate the relationship between atrial remodeling, AF and the gene of interest. The TAC model seems to be a clinically relevant model as it mimics pressure overload as seen with hypertension or heart failure, and these diseases are often associated with atrial remodeling underlying AF.

The ventricular pressure overload model, as used in this study is suitable to investigate the effect on atrial hypertrophy and inflammation, but cannot be used to investigate the effect on inducibility of AF.

## Limitations

The lack of fibrosis might be due to the short time frame of our study. It is possible that after a longer period of TAC, fibrosis might develop also in the atria as fibrotic gene expression is already slightly but significantly increased at eight weeks pressure-overload. A later time point, however, would be confounded by the development of heart failure as LV function is starting to deteriorate after eight weeks. It would be difficult to separate pressure-dependent effects in the atria from other factors such as neuro-hormonal activation, which was the goal of the present study.

A surprising finding was the lack of AF inducibility that we explain by the lack of fibrosis or of pronounced changes related to electrical remodeling. On the other hand, we found a surprisingly high AF inducibility in the sham group that might be explained by the fact that these mice also underwent thoracotomy. We have no control group with no surgery to rule out that sham surgery induces AF inducibility *per se*. Expression data were obtained from pooled RA and LA, although we realize that the RA and LA might respond different to disease [32]–[34], we feel that this does not affect our results. Most likely the RA has remodeled less, and we are underestimating the observed changes in gene expression.

## Conclusions

Permanent ventricular pressure overload by TAC induced atrial remodeling, including hypertrophy, dilatation and inflammation, but no detectable fibrosis. Atrial remodeling was associated with an increase in left ventricular end diastolic pressure, but not with AF induction. In summary, our results indicate that this model of atrial pressure-overload cannot be used to investigate AF inducibility, but can be used to investigate atrial remodeling.
